# Supplementary material for: Anti-Inflammatory and Autophagy Activation Effects of 7-Methylsulfonylheptyl Isothiocyanate Could Suppress Skin Aging: In Vitro Evidence
Source: Antioxidants (Basel). 2024 Oct 23;13(11):1282. doi: 10.3390/antiox13111282 (PMC11591029; doi:10.3390/antiox13111282)
Supplement: Supplementary file 1 [file antioxidants-13-01282-s001.zip › antioxidants-3093545-supplementary.pdf]

Table S1. Gene primer used for RT-PCR.

Gene		Primer sequences
IL-1β	forward	5′-TGTGAAATGCCACCTTTTGA-3′
	reverse	5′-GTAGCTGCCACAGCTTCTCC-3′
IL-6	forward	5′-AATTTCCTCTGGTCTTC- TGG-3′
	reverse	5′-TAGCCACTCCTTCTGTGACTC-3′
COX-2	forward	5′-CAGCAAATCCTTGCTGTTCC-3′
	reverse	5′-CCATCCTTGAAAAGGCGCAG-3′
PGEs	forward	5′-ATGCCTTCCCCGGGCCTG-3′
	reverse	5′-TCACAGATGGTGGGCCAC-3′
NLRP3	forward	5′-CCTGACCCAAACCCACCAGT-3′
	reverse	5′-TTCTTTCGGATGAGGCTGCTTA-3′
ASC	forward	5′-TGAGCAGCTGCAAACGACTA-3′
	reverse	5′-ACACTGCCATGCAAAGCATC-3′
Caspase-1	forward	5′- ATGAATCACCAACACCAG-3′
	reverse	5′- CTTGACGCATCCTAATCC-3′
GAPDH	forward	5′-TCAGCAATGCATCCTGCACCAC-3′
	reverse	5′-TGCCAGTGAGCTTCCCGTTCAG-3′

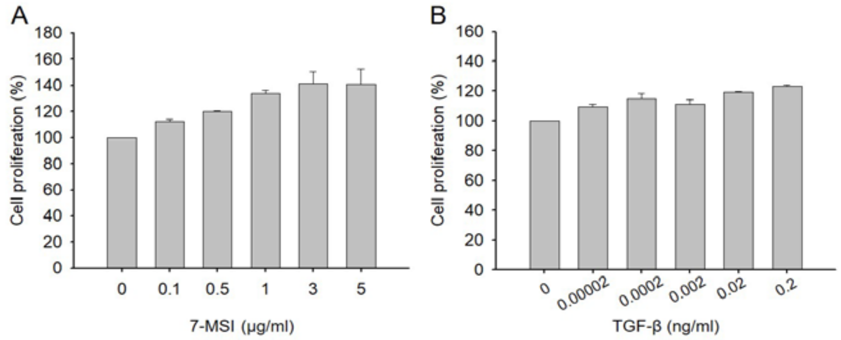

Supplementary Figure S1. Effect of 7-MSI on Cell Viability in RAW 264.7 Cells. RAW 264.7 cells underwent treatment with incremental concentrations of 7-MSI (0 - 5 μg/ml) (Panel A) and TGF-β (0 - 0.2 ng/ml) (Panel B) over a duration of 24 hours. Cell viability was determined through an MTS assay following the procedures outlined in the CellTiter 96® AQueous Non-Radioactive Cell Proliferation Assay (Promega, USA). TGF-β was employed as a positive control in this study.
